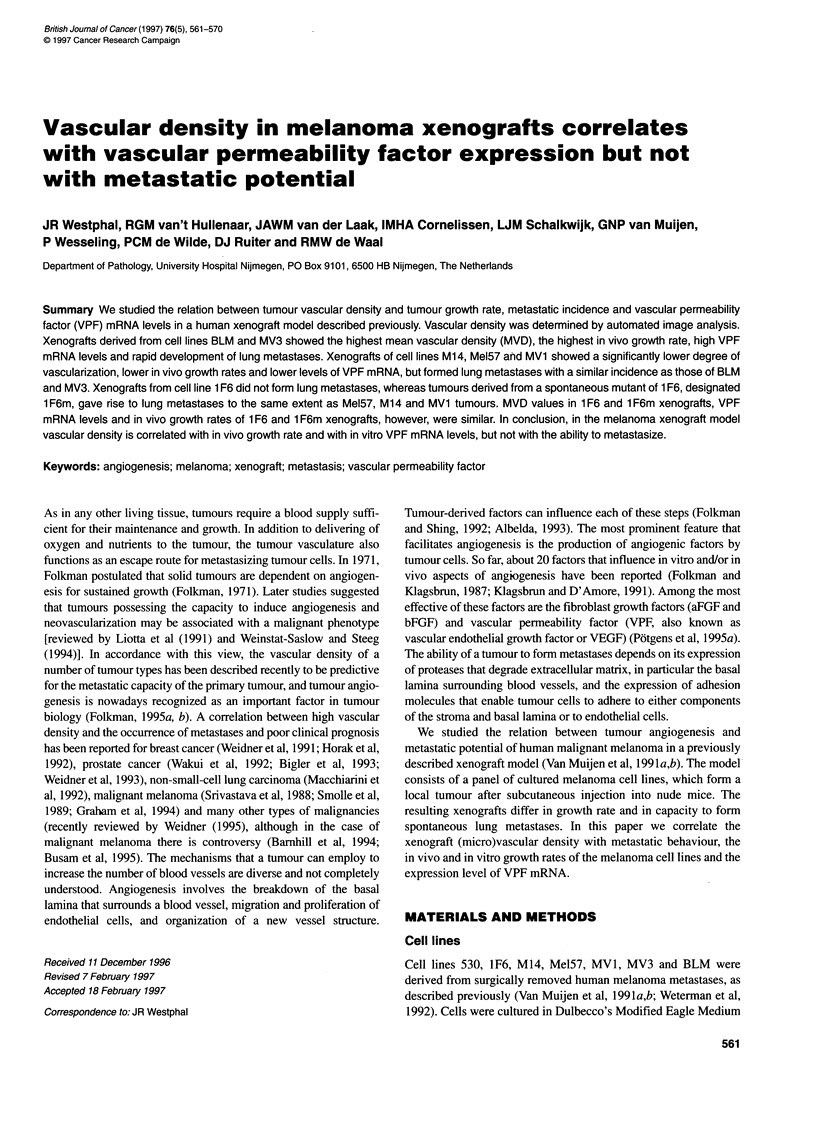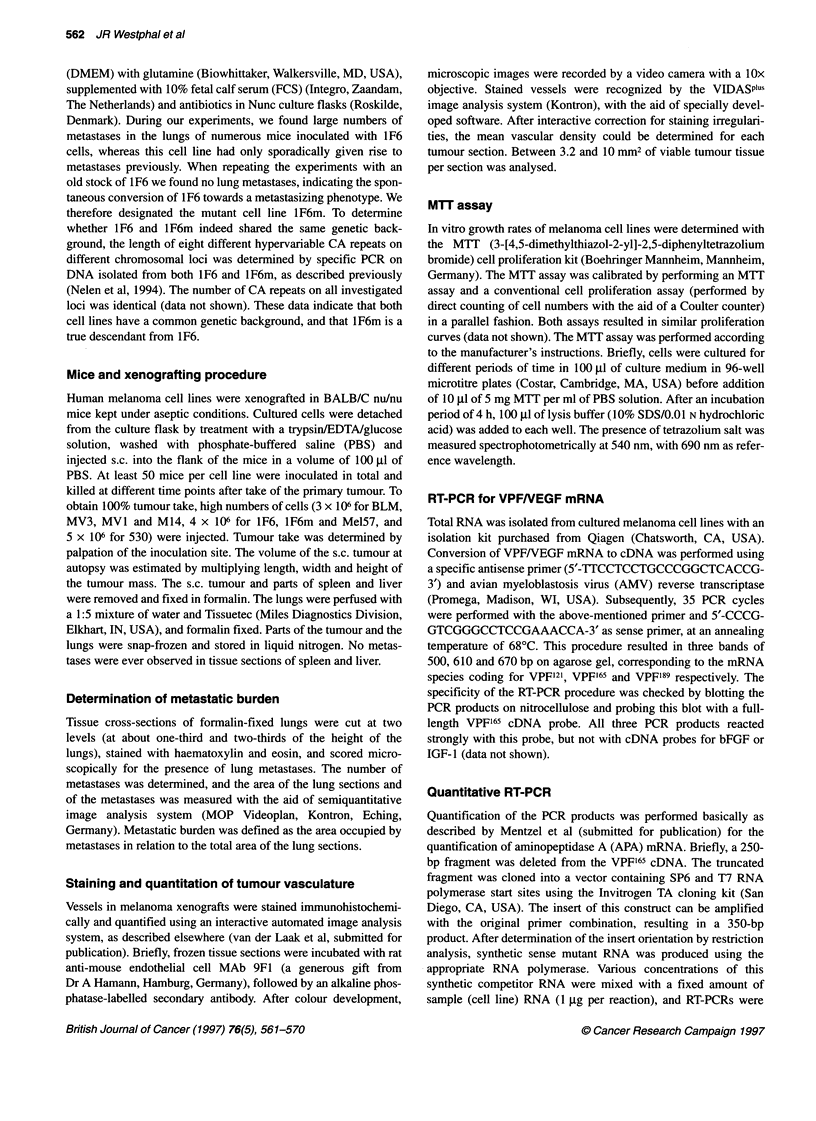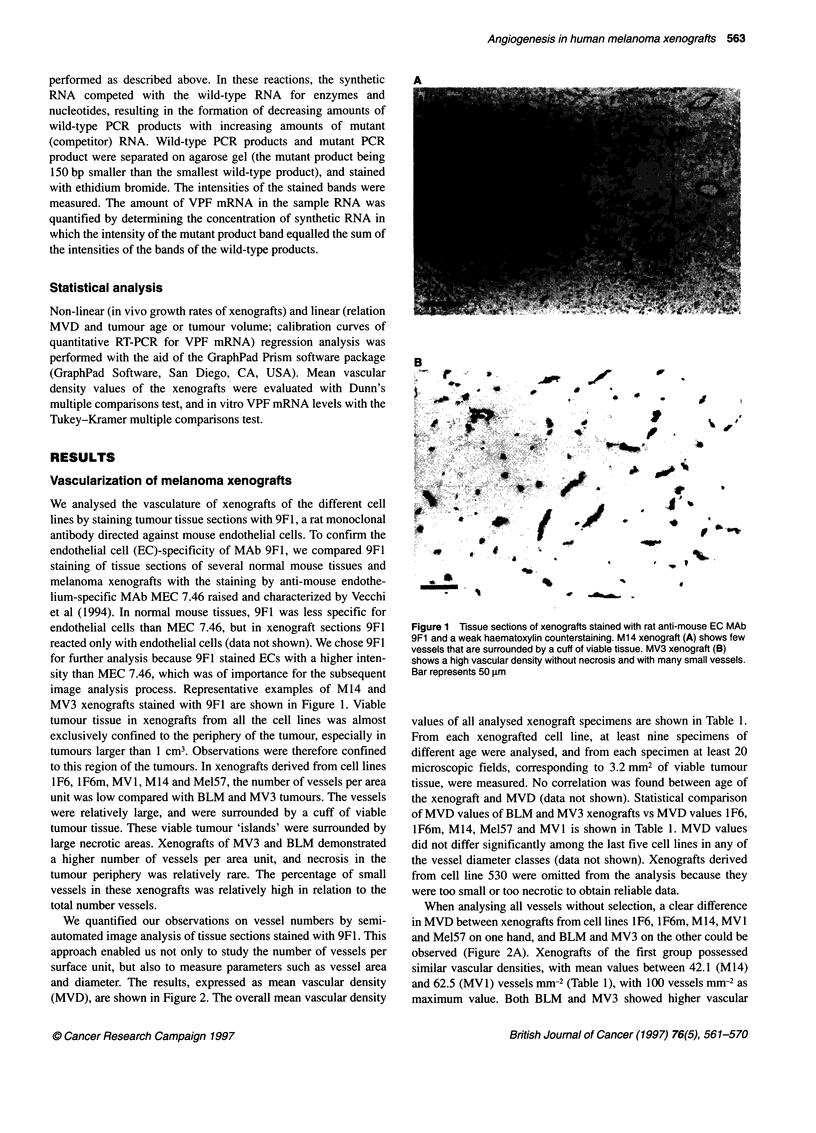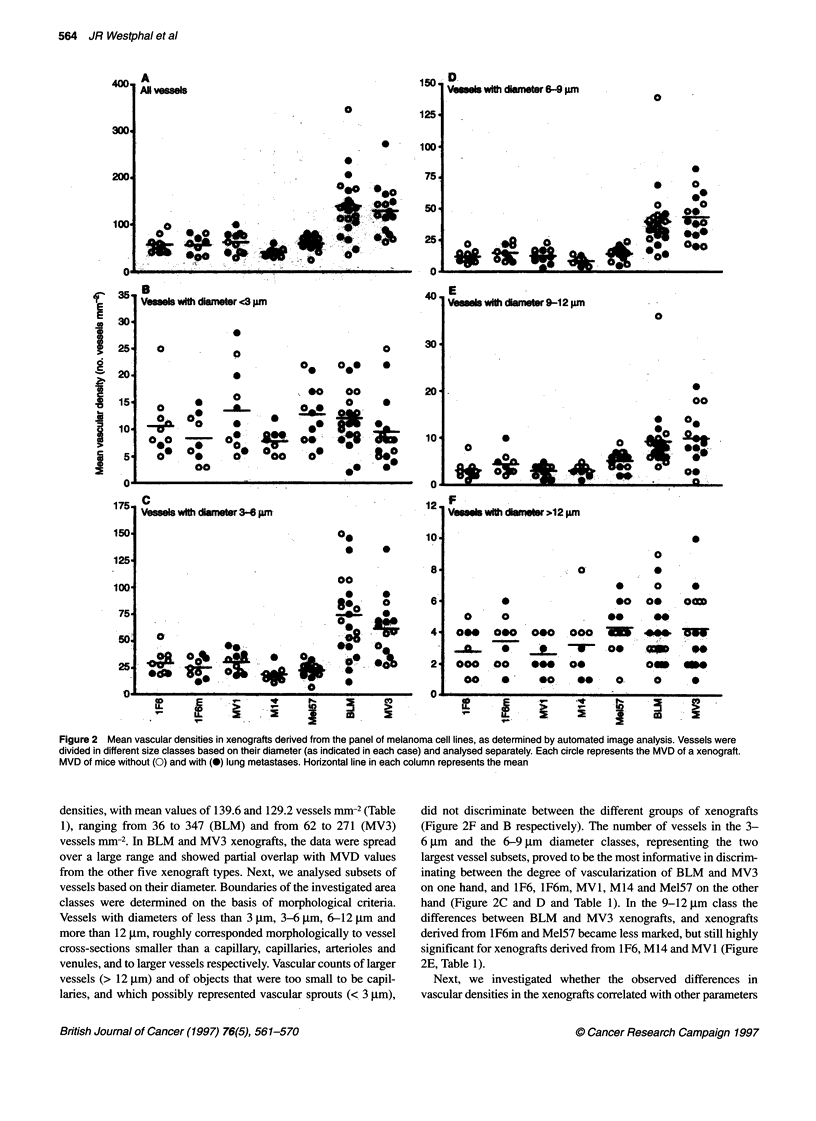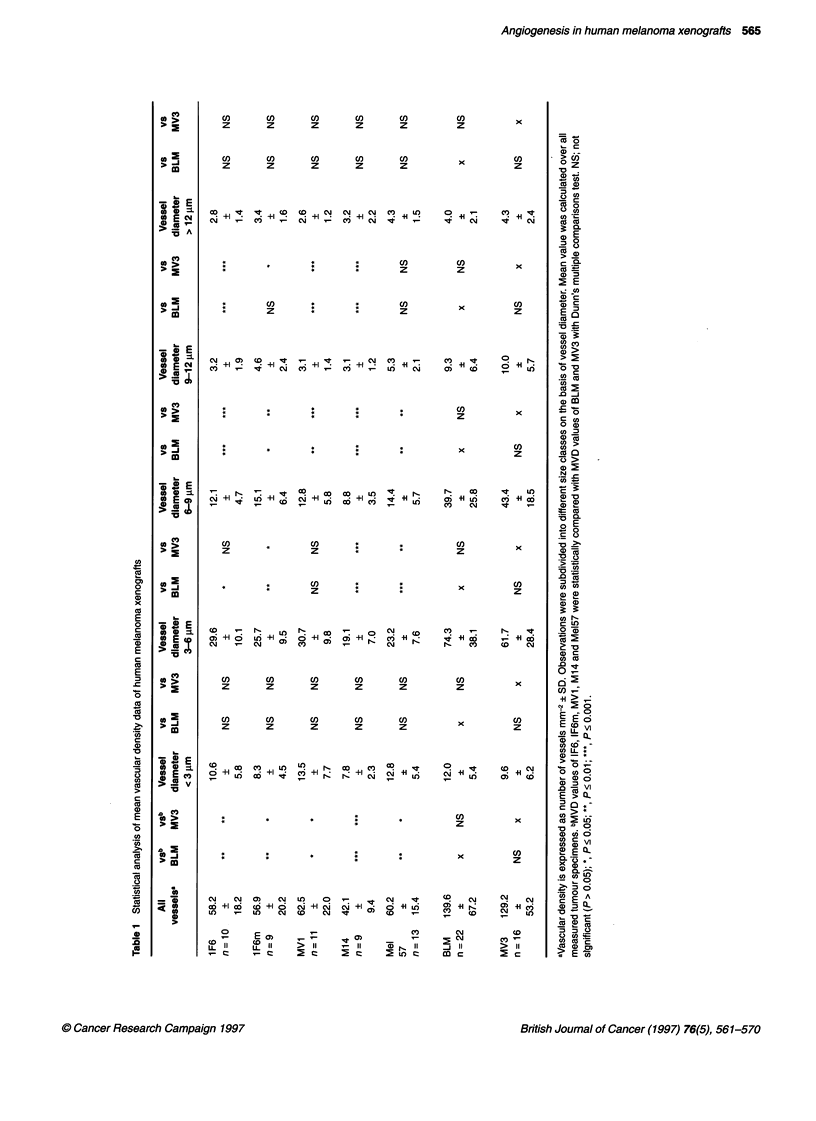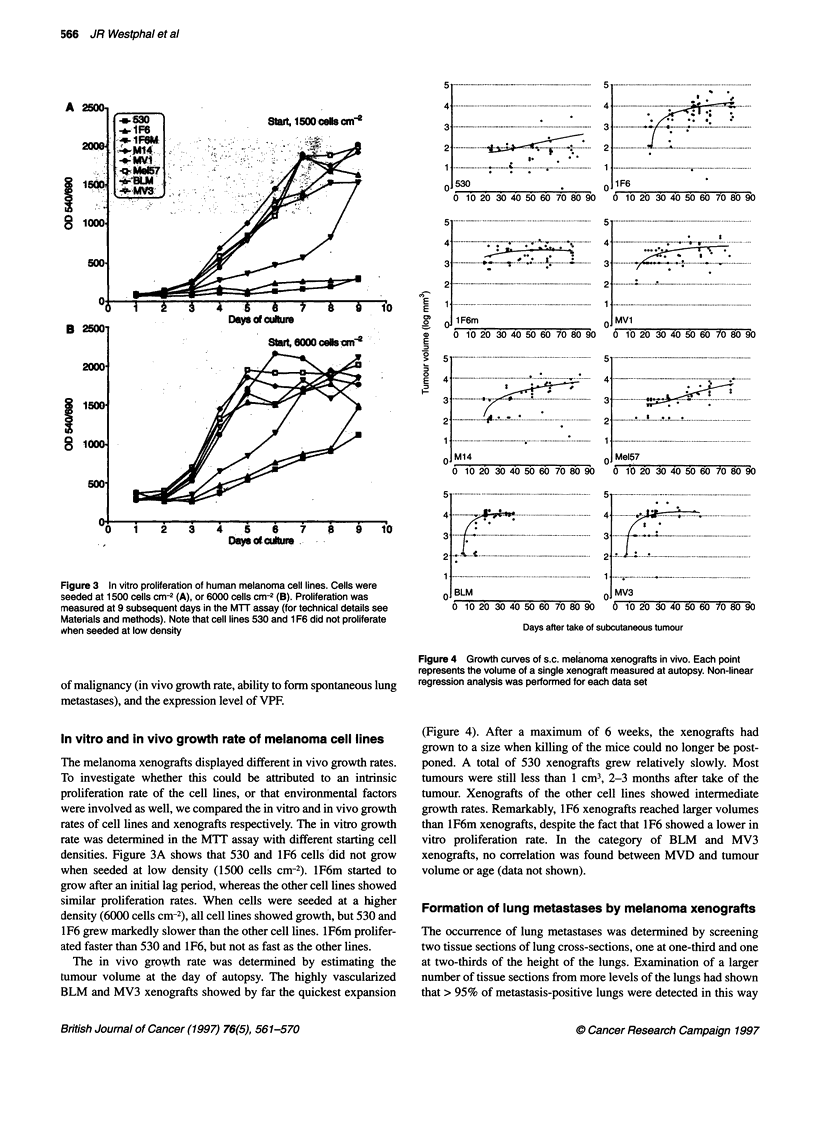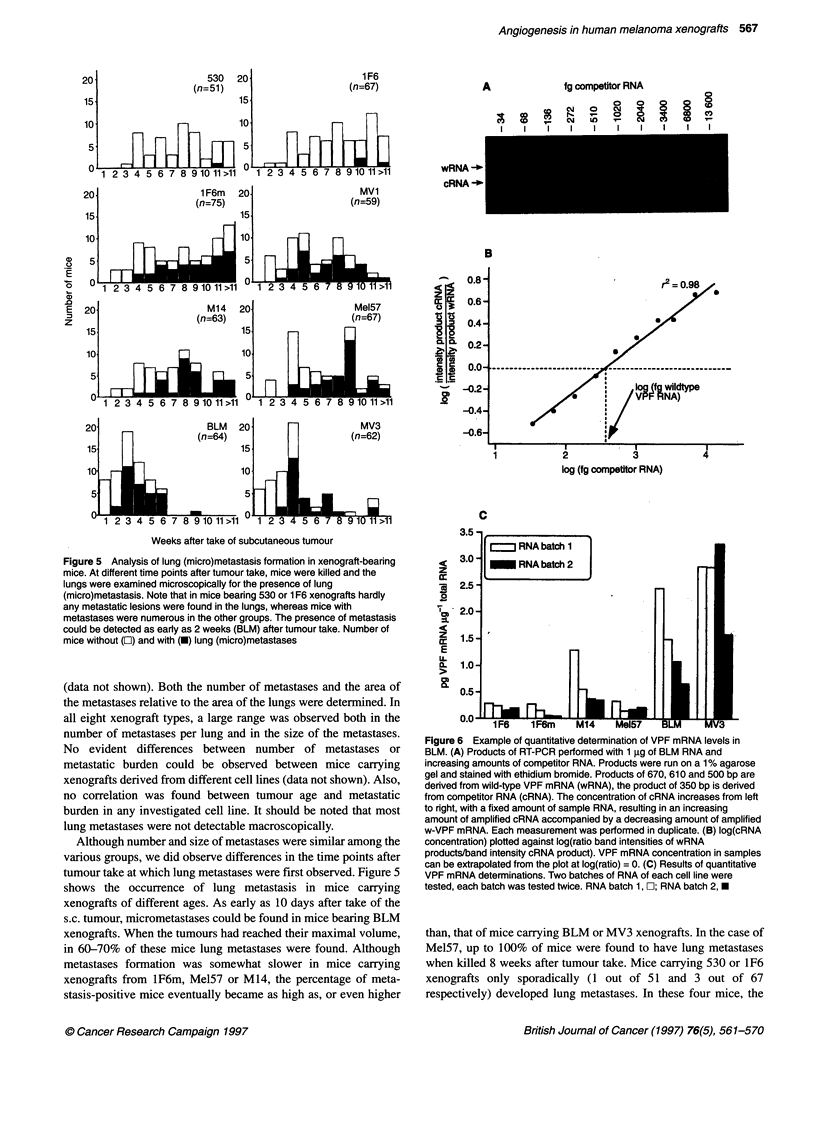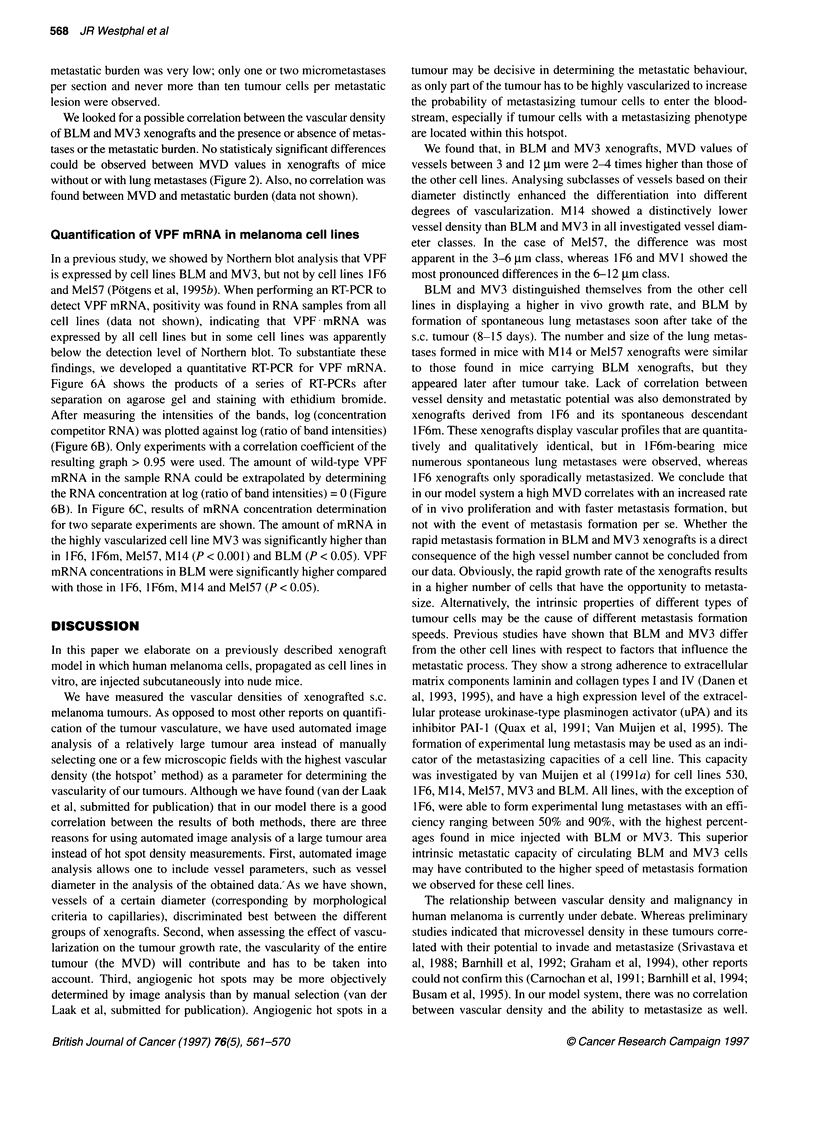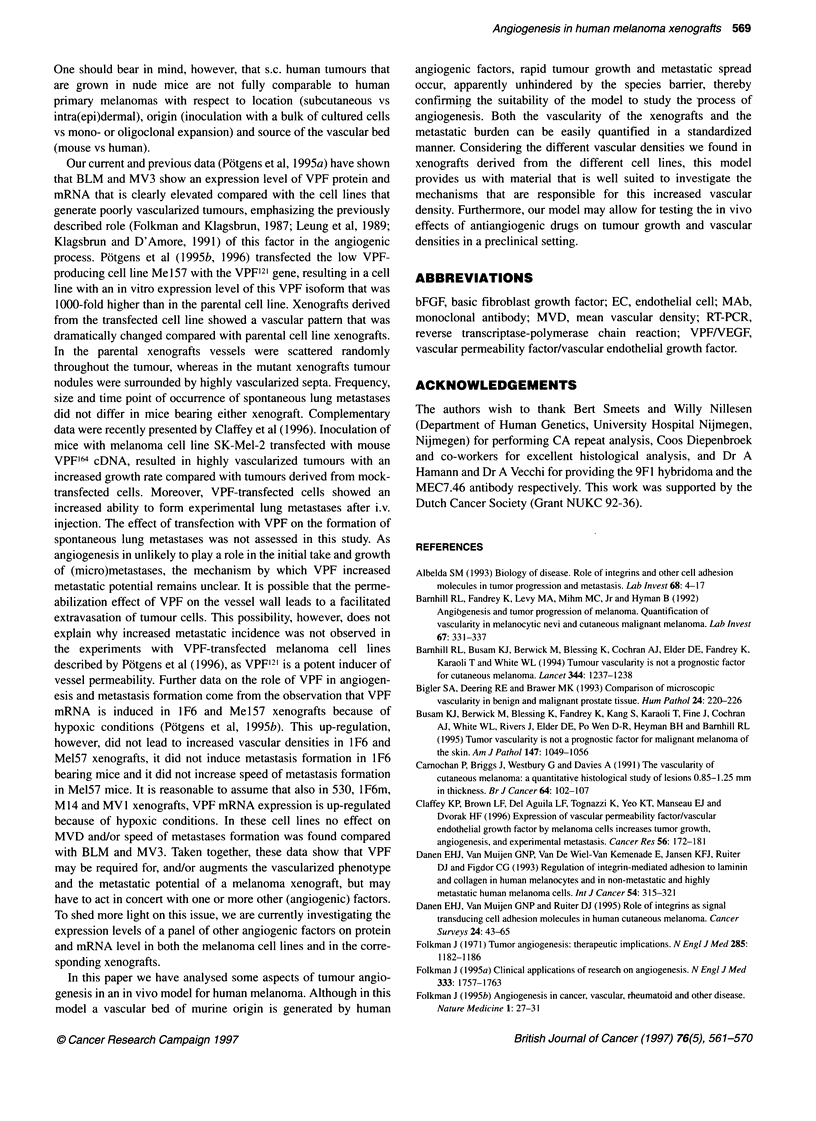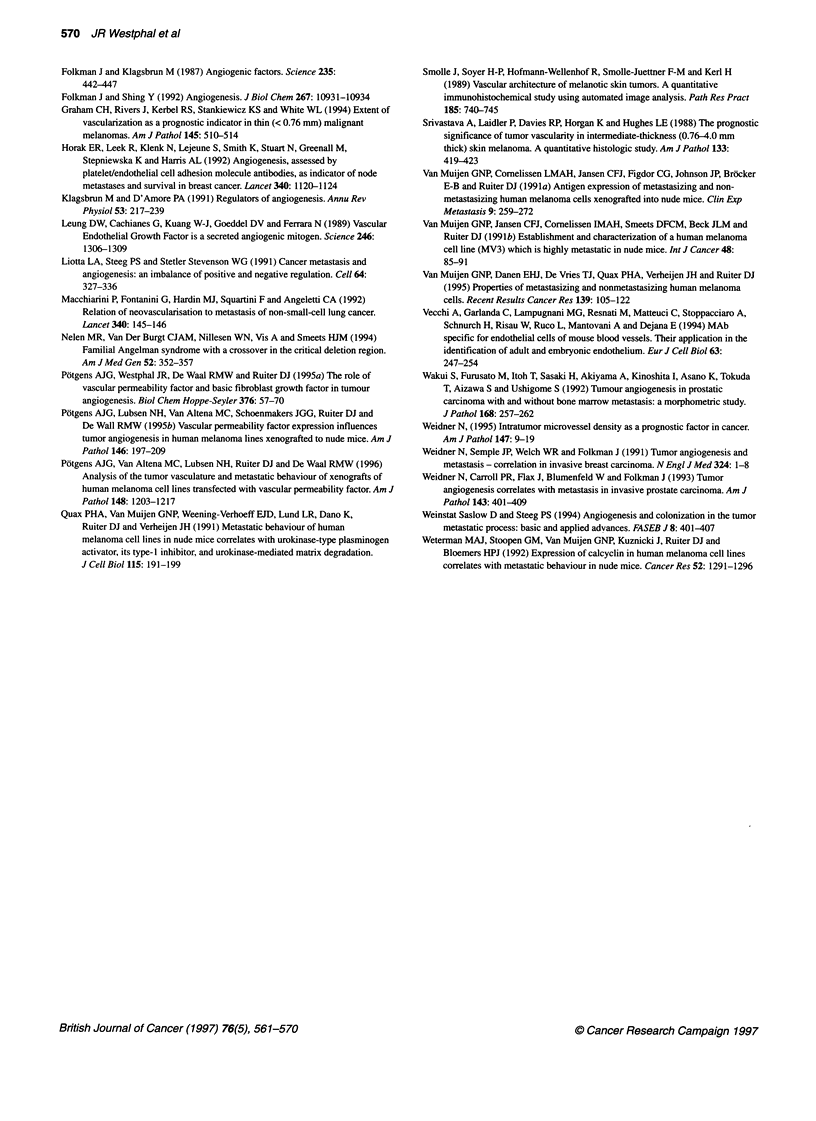# Vascular density in melanoma xenografts correlates with vascular permeability factor expression but not with metastatic potential.

**DOI:** 10.1038/bjc.1997.427

**Published:** 1997

**Authors:** J. R. Westphal, R. G. van't Hullenaar, J. A. van der Laak, I. M. Cornelissen, L. J. Schalkwijk, G. N. van Muijen, P. Wesseling, P. C. de Wilde, D. J. Ruiter, R. M. de Waal

**Affiliations:** Department of Pathology, University Hospital Nijmegen, The Netherlands.

## Abstract

**Images:**


British Journal of Cancer (1997) 76(5), 561–570
© 1997 Cancer Research Campaign

### Vascular density in melanoma xenografts correlates with vascular permeability factor expression but not with metastatic potential

**JR Westphal, RGM van't Hullenaar, JAWM van der Laak, IMHA Cornelissen, LJM Schalkwijk, GNP van Muijen, P Wesseling, PCM de Wilde, DJ Ruiter and RMW de Waal**

Department of Pathology, University Hospital Nijmegen, PO Box 9101, 6500 HB Nijmegen, The Netherlands

**Summary** We studied the relation between tumour vascular density and tumour growth rate, metastatic incidence and vascular permeability factor (VPF) mRNA levels in a human xenograft model described previously. Vascular density was determined by automated image analysis. Xenografts derived from cell lines BLM and MV3 showed the highest mean vascular density (MVD), the highest in vivo growth rate, high VPF mRNA levels and rapid development of lung metastases. Xenografts of cell lines M14, Mel57 and MV1 showed a significantly lower degree of vascularization, lower in vivo growth rates and lower levels of VPF mRNA, but formed lung metastases with a similar incidence as those of BLM and MV3. Xenografts from cell line 1F6 did not form lung metastases, whereas tumours derived from a spontaneous mutant of 1F6, designated 1F6m, gave rise to lung metastases to the same extent as Mel57, M14 and MV1 tumours. MVD values in 1F6 and 1F6m xenografts, VPF mRNA levels and in vivo growth rates of 1F6 and 1F6m xenografts, however, were similar. In conclusion, in the melanoma xenograft model vascular density is correlated with in vivo growth rate and with in vitro VPF mRNA levels, but not with the ability to metastasize.

**Keywords:** angiogenesis; melanoma; xenograft; metastasis; vascular permeability factor

As in any other living tissue, tumours require a blood supply sufficient for their maintenance and growth. In addition to delivering of oxygen and nutrients to the tumour, the tumour vasculature also functions as an escape route for metastasizing tumour cells. In 1971, Folkman postulated that solid tumours are dependent on angiogenesis for sustained growth (Folkman, 1971). Later studies suggested that tumours possessing the capacity to induce angiogenesis and neovascularization may be associated with a malignant phenotype [reviewed by Liotta et al (1991) and Weinstat-Saslow and Steeg (1994)]. In accordance with this view, the vascular density of a number of tumour types has been described recently to be predictive for the metastatic capacity of the primary tumour, and tumour angiogenesis is nowadays recognized as an important factor in tumour biology (Folkman, 1995*a, b*). A correlation between high vascular density and the occurrence of metastases and poor clinical prognosis has been reported for breast cancer (Weidner et al, 1991; Horak et al, 1992), prostate cancer (Wakui et al, 1992; Bigler et al, 1993; Weidner et al, 1993), non-small-cell lung carcinoma (Macchiarini et al, 1992), malignant melanoma (Srivastava et al, 1988; Smolle et al, 1989; Graham et al, 1994) and many other types of malignancies (recently reviewed by Weidner (1995), although in the case of malignant melanoma there is controversy (Barnhill et al, 1994; Busam et al, 1995). The mechanisms that a tumour can employ to increase the number of blood vessels are diverse and not completely understood. Angiogenesis involves the breakdown of the basal lamina that surrounds a blood vessel, migration and proliferation of endothelial cells, and organization of a new vessel structure. Tumour-derived factors can influence each of these steps (Folkman and Shing, 1992; Albelda, 1993). The most prominent feature that facilitates angiogenesis is the production of angiogenic factors by tumour cells. So far, about 20 factors that influence in vitro and/or in vivo aspects of angiogenesis have been reported (Folkman and Klagsbrun, 1987; Klagsbrun and D'Amore, 1991). Among the most effective of these factors are the fibroblast growth factors (aFGF and bFGF) and vascular permeability factor (VPF, also known as vascular endothelial growth factor or VEGF) (Pötgens et al, 1995*a*). The ability of a tumour to form metastases depends on its expression of proteases that degrade extracellular matrix, in particular the basal lamina surrounding blood vessels, and the expression of adhesion molecules that enable tumour cells to adhere to either components of the stroma and basal lamina or to endothelial cells.

We studied the relation between tumour angiogenesis and metastatic potential of human malignant melanoma in a previously described xenograft model (Van Muijen et al, 1991*a,b*). The model consists of a panel of cultured melanoma cell lines, which form a local tumour after subcutaneous injection into nude mice. The resulting xenografts differ in growth rate and in capacity to form spontaneous lung metastases. In this paper we correlate the xenograft (micro)vascular density with metastatic behaviour, the in vivo and in vitro growth rates of the melanoma cell lines and the expression level of VPF mRNA.

*Received 11 December 1996*
*Revised 7 February 1997*
*Accepted 18 February 1997*

*Correspondence to:* JR Westphal

### MATERIALS AND METHODS

### Cell lines

Cell lines 530, 1F6, M14, Mel57, MV1, MV3 and BLM were derived from surgically removed human melanoma metastases, as described previously (Van Muijen et al, 1991*a,b*; Weterman et al, 1992). Cells were cultured in Dulbecco's Modified Eagle Medium

(DMEM) with glutamine (Biowhittaker, Walkersville, MD, USA), supplemented with 10% fetal calf serum (FCS) (Integro, Zaandam, The Netherlands) and antibiotics in Nunc culture flasks (Roskilde, Denmark). During our experiments, we found large numbers of metastases in the lungs of numerous mice inoculated with 1F6 cells, whereas this cell line had only sporadically given rise to metastases previously. When repeating the experiments with an old stock of 1F6 we found no lung metastases, indicating the spontaneous conversion of 1F6 towards a metastasizing phenotype. We therefore designated the mutant cell line 1F6m. To determine whether 1F6 and 1F6m indeed shared the same genetic background, the length of eight different hypervariable CA repeats on different chromosomal loci was determined by specific PCR on DNA isolated from both 1F6 and 1F6m, as described previously (Nelen et al, 1994). The number of CA repeats on all investigated loci was identical (data not shown). These data indicate that both cell lines have a common genetic background, and that 1F6m is a true descendant from 1F6.

### Mice and xenografting procedure

Human melanoma cell lines were xenografted in BALB/C nu/nu mice kept under aseptic conditions. Cultured cells were detached from the culture flask by treatment with a trypsin/EDTA/glucose solution, washed with phosphate-buffered saline (PBS) and injected s.c. into the flank of the mice in a volume of 100 μl of PBS. At least 50 mice per cell line were inoculated in total and killed at different time points after take of the primary tumour. To obtain 100% tumour take, high numbers of cells ($3 \times 10^6$ for BLM, MV3, MV1 and M14, $4 \times 10^6$ for 1F6, 1F6m and Mel57, and $5 \times 10^6$ for 530) were injected. Tumour take was determined by palpation of the inoculation site. The volume of the s.c. tumour at autopsy was estimated by multiplying length, width and height of the tumour mass. The s.c. tumour and parts of spleen and liver were removed and fixed in formalin. The lungs were perfused with a 1:5 mixture of water and Tissuetec (Miles Diagnostics Division, Elkhart, IN, USA), and formalin fixed. Parts of the tumour and the lungs were snap-frozen and stored in liquid nitrogen. No metastases were ever observed in tissue sections of spleen and liver.

### Determination of metastatic burden

Tissue cross-sections of formalin-fixed lungs were cut at two levels (at about one-third and two-thirds of the height of the lungs), stained with haematoxylin and eosin, and scored microscopically for the presence of lung metastases. The number of metastases was determined, and the area of the lung sections and of the metastases was measured with the aid of semiquantitative image analysis system (MOP Videoplan, Kontron, Eching, Germany). Metastatic burden was defined as the area occupied by metastases in relation to the total area of the lung sections.

### Staining and quantitation of tumour vasculature

Vessels in melanoma xenografts were stained immunohistochemically and quantified using an interactive automated image analysis system, as described elsewhere (van der Laak et al, submitted for publication). Briefly, frozen tissue sections were incubated with rat anti-mouse endothelial cell MAb 9F1 (a generous gift from Dr A Hamann, Hamburg, Germany), followed by an alkaline phosphatase-labelled secondary antibody. After colour development, microscopic images were recorded by a video camera with a 10× objective. Stained vessels were recognized by the VIDAS$^{plus}$ image analysis system (Kontron), with the aid of specially developed software. After interactive correction for staining irregularities, the mean vascular density could be determined for each tumour section. Between 3.2 and 10 $mm^2$ of viable tumour tissue per section was analysed.

### MTT assay

In vitro growth rates of melanoma cell lines were determined with the MTT (3-[4,5-dimethylthiazol-2-yl]-2,5-diphenyltetrazolium bromide) cell proliferation kit (Boehringer Mannheim, Mannheim, Germany). The MTT assay was calibrated by performing an MTT assay and a conventional cell proliferation assay (performed by direct counting of cell numbers with the aid of a Coulter counter) in a parallel fashion. Both assays resulted in similar proliferation curves (data not shown). The MTT assay was performed according to the manufacturer's instructions. Briefly, cells were cultured for different periods of time in 100 μl of culture medium in 96-well microtitre plates (Costar, Cambridge, MA, USA) before addition of 10 μl of 5 mg MTT per ml of PBS solution. After an incubation period of 4 h, 100 μl of lysis buffer (10% SDS/0.01 N hydrochloric acid) was added to each well. The presence of tetrazolium salt was measured spectrophotometrically at 540 nm, with 690 nm as reference wavelength.

### RT-PCR for VPF/VEGF mRNA

Total RNA was isolated from cultured melanoma cell lines with an isolation kit purchased from Qiagen (Chatsworth, CA, USA). Conversion of VPF/VEGF mRNA to cDNA was performed using a specific antisense primer (5′-TTCCTCCTGCCCGGCTCACCG-3′) and avian myeloblastosis virus (AMV) reverse transcriptase (Promega, Madison, WI, USA). Subsequently, 35 PCR cycles were performed with the above-mentioned primer and 5′-CCCG-GTCGGGCCTCCGAAACCA-3′ as sense primer, at an annealing temperature of 68°C. This procedure resulted in three bands of 500, 610 and 670 bp on agarose gel, corresponding to the mRNA species coding for $VPF^{121}$, $VPF^{165}$ and $VPF^{189}$ respectively. The specificity of the RT-PCR procedure was checked by blotting the PCR products on nitrocellulose and probing this blot with a full-length $VPF^{165}$ cDNA probe. All three PCR products reacted strongly with this probe, but not with cDNA probes for bFGF or IGF-1 (data not shown).

### Quantitative RT-PCR

Quantification of the PCR products was performed basically as described by Mentzel et al (submitted for publication) for the quantification of aminopeptidase A (APA) mRNA. Briefly, a 250-bp fragment was deleted from the $VPF^{165}$ cDNA. The truncated fragment was cloned into a vector containing SP6 and T7 RNA polymerase start sites using the Invitrogen TA cloning kit (San Diego, CA, USA). The insert of this construct can be amplified with the original primer combination, resulting in a 350-bp product. After determination of the insert orientation by restriction analysis, synthetic sense mutant RNA was produced using the appropriate RNA polymerase. Various concentrations of this synthetic competitor RNA were mixed with a fixed amount of sample (cell line) RNA (1 μg per reaction), and RT-PCRs were

© Cancer Research Campaign 1997

performed as described above. In these reactions, the synthetic RNA competed with the wild-type RNA for enzymes and nucleotides, resulting in the formation of decreasing amounts of wild-type PCR products with increasing amounts of mutant (competitor) RNA. Wild-type PCR products and mutant PCR product were separated on agarose gel (the mutant product being 150 bp smaller than the smallest wild-type product), and stained with ethidium bromide. The intensities of the stained bands were measured. The amount of VPF mRNA in the sample RNA was quantified by determining the concentration of synthetic RNA in which the intensity of the mutant product band equalled the sum of the intensities of the bands of the wild-type products.

### Statistical analysis

Non-linear (in vivo growth rates of xenografts) and linear (relation MVD and tumour age or tumour volume; calibration curves of quantitative RT-PCR for VPF mRNA) regression analysis was performed with the aid of the GraphPad Prism software package (GraphPad Software, San Diego, CA, USA). Mean vascular density values of the xenografts were evaluated with Dunn's multiple comparisons test, and in vitro VPF mRNA levels with the Tukey–Kramer multiple comparisons test.

### RESULTS

### Vascularization of melanoma xenografts

We analysed the vasculature of xenografts of the different cell lines by staining tumour tissue sections with 9F1, a rat monoclonal antibody directed against mouse endothelial cells. To confirm the endothelial cell (EC)-specificity of MAb 9F1, we compared 9F1 staining of tissue sections of several normal mouse tissues and melanoma xenografts with the staining by anti-mouse endothelium-specific MAb MEC 7.46 raised and characterized by Vecchi et al (1994). In normal mouse tissues, 9F1 was less specific for endothelial cells than MEC 7.46, but in xenograft sections 9F1 reacted only with endothelial cells (data not shown). We chose 9F1 for further analysis because 9F1 stained ECs with a higher intensity than MEC 7.46, which was of importance for the subsequent image analysis process. Representative examples of M14 and MV3 xenografts stained with 9F1 are shown in Figure 1. Viable tumour tissue in xenografts from all the cell lines was almost exclusively confined to the periphery of the tumour, especially in tumours larger than 1 $cm^3$. Observations were therefore confined to this region of the tumours. In xenografts derived from cell lines 1F6, 1F6m, MV1, M14 and Mel57, the number of vessels per area unit was low compared with BLM and MV3 tumours. The vessels were relatively large, and were surrounded by a cuff of viable tumour tissue. These viable tumour 'islands' were surrounded by large necrotic areas. Xenografts of MV3 and BLM demonstrated a higher number of vessels per area unit, and necrosis in the tumour periphery was relatively rare. The percentage of small vessels in these xenografts was relatively high in relation to the total number vessels.

We quantified our observations on vessel numbers by semi-automated image analysis of tissue sections stained with 9F1. This approach enabled us not only to study the number of vessels per surface unit, but also to measure parameters such as vessel area and diameter. The results, expressed as mean vascular density (MVD), are shown in Figure 2. The overall mean vascular density

**Figure 1** Tissue sections of xenografts stained with rat anti-mouse EC MAb 9F1 and a weak haematoxylin counterstaining. M14 xenograft (**A**) shows few vessels that are surrounded by a cuff of viable tissue. MV3 xenograft (**B**) shows a high vascular density without necrosis and with many small vessels. Bar represents 50 μm values of all analysed xenograft specimens are shown in Table 1. From each xenografted cell line, at least nine specimens of different age were analysed, and from each specimen at least 20 microscopic fields, corresponding to 3.2 $mm^2$ of viable tumour tissue, were measured. No correlation was found between age of the xenograft and MVD (data not shown). Statistical comparison of MVD values of BLM and MV3 xenografts vs MVD values 1F6, 1F6m, M14, Mel57 and MV1 is shown in Table 1. MVD values did not differ significantly among the last five cell lines in any of the vessel diameter classes (data not shown). Xenografts derived from cell line 530 were omitted from the analysis because they were too small or too necrotic to obtain reliable data.

When analysing all vessels without selection, a clear difference in MVD between xenografts from cell lines 1F6, 1F6m, M14, MV1 and Mel57 on one hand, and BLM and MV3 on the other could be observed (Figure 2A). Xenografts of the first group possessed similar vascular densities, with mean values between 42.1 (M14) and 62.5 (MV1) vessels $mm^{-2}$ (Table 1), with 100 vessels $mm^{-2}$ as maximum value. Both BLM and MV3 showed higher vascular

© Cancer Research Campaign 1997

**Figure 2** Mean vascular densities in xenografts derived from the panel of melanoma cell lines, as determined by automated image analysis. Vessels were divided in different size classes based on their diameter (as indicated in each case) and analysed separately. Each circle represents the MVD of a xenograft. MVD of mice without (○) and with (●) lung metastases. Horizontal line in each column represents the mean densities, with mean values of 139.6 and 129.2 vessels mm$^{-2}$ (Table 1), ranging from 36 to 347 (BLM) and from 62 to 271 (MV3) vessels mm$^{-2}$. In BLM and MV3 xenografts, the data were spread over a large range and showed partial overlap with MVD values from the other five xenograft types. Next, we analysed subsets of vessels based on their diameter. Boundaries of the investigated area classes were determined on the basis of morphological criteria. Vessels with diameters of less than 3 μm, 3–6 μm, 6–12 μm and more than 12 μm, roughly corresponded morphologically to vessel cross-sections smaller than a capillary, capillaries, arterioles and venules, and to larger vessels respectively. Vascular counts of larger vessels (> 12 μm) and of objects that were too small to be capillaries, and which possibly represented vascular sprouts (< 3 μm), did not discriminate between the different groups of xenografts (Figure 2F and B respectively). The number of vessels in the 3–6 μm and the 6–9 μm diameter classes, representing the two largest vessel subsets, proved to be the most informative in discriminating between the degree of vascularization of BLM and MV3 on one hand, and 1F6, 1F6m, MV1, M14 and Mel57 on the other hand (Figure 2C and D and Table 1). In the 9–12 μm class the differences between BLM and MV3 xenografts, and xenografts derived from 1F6m and Mel57 became less marked, but still highly significant for xenografts derived from 1F6, M14 and MV1 (Figure 2E, Table 1).

Next, we investigated whether the observed differences in vascular densities in the xenografts correlated with other parameters

© Cancer Research Campaign 1997

**Table 1** Statistical analysis of mean vascular density data of human melanoma xenografts

| | All vessels[a] | vs[b] BLM | vs[b] MV3 | Vessel diameter < 3 μm | vs BLM | vs MV3 | Vessel diameter 3–6 μm | vs BLM | vs MV3 | Vessel diameter 6–9 μm | vs BLM | vs MV3 | Vessel diameter 9–12 μm | vs BLM | vs MV3 | Vessel diameter > 12 μm | vs BLM | vs MV3 |
|---|---|---|---|---|---|---|---|---|---|---|---|---|---|---|---|---|---|---|
| 1F6 $n$ = 10 | 58.2 ± 18.2 | ** | ** | 10.6 ± 5.8 | NS | NS | 29.6 ± 10.1 | * | NS | 12.1 ± 4.7 | *** | *** | 3.2 ± 1.9 | *** | *** | 2.8 ± 1.4 | NS | NS |
| 1F6m $n$ = 9 | 56.9 ± 20.2 | ** | * | 8.3 ± 4.5 | NS | NS | 25.7 ± 9.5 | ** | * | 15.1 ± 6.4 | * | ** | 4.6 ± 2.4 | NS | * | 3.4 ± 1.6 | NS | NS |
| MV1 $n$ = 11 | 62.5 ± 22.0 | * | * | 13.5 ± 7.7 | NS | NS | 30.7 ± 9.8 | NS | NS | 12.8 ± 5.8 | ** | *** | 3.1 ± 1.4 | *** | *** | 2.6 ± 1.2 | NS | NS |
| M14 $n$ = 9 | 42.1 ± 9.4 | *** | *** | 7.8 ± 2.3 | NS | NS | 19.1 ± 7.0 | *** | *** | 8.8 ± 3.5 | *** | *** | 3.1 ± 1.2 | *** | *** | 3.2 ± 2.2 | NS | NS |
| Mel 57 $n$ = 13 | 60.2 ± 15.4 | ** | * | 12.8 ± 5.4 | NS | NS | 23.2 ± 7.6 | *** | ** | 14.4 ± 5.7 | ** | ** | 5.3 ± 2.1 | NS | NS | 4.3 ± 1.5 | NS | NS |
| BLM n = 22 | 139.6 ± 67.2 | x | NS | 12.0 ± 5.4 | x | NS | 74.3 ± 38.1 | x | NS | 39.7 ± 25.8 | x | NS | 9.3 ± 6.4 | x | NS | 4.0 ± 2.1 | x | NS |
| MV3 n = 16 | 129.2 ± 53.2 | NS | x | 9.6 ± 6.2 | NS | x | 61.7 ± 28.4 | NS | x | 43.4 ± 18.5 | NS | x | 10.0 ± 5.7 | NS | x | 4.3 ± 2.4 | NS | x |

[a]Vascular density is expressed as number of vessels $mm^{-2}$ ± SD. Observations were subdivided into different size classes on the basis of vessel diameter. Mean value was calculated over all measured tumour specimens. [b]MVD values of IF6, IF6m, MV1, M14 and Mel57 were statistically compared with MVD values of BLM and MV3 with Dunn's multiple comparisons test. NS; not significant ($P > 0.05$); *, $P \leq 0.05$; **, $P \leq 0.01$; ***, $P \leq 0.001$.

© Cancer Research Campaign 1997

**Figure 3** In vitro proliferation of human melanoma cell lines. Cells were seeded at 1500 cells cm$^{-2}$ (**A**), or 6000 cells cm$^{-2}$ (**B**). Proliferation was measured at 9 subsequent days in the MTT assay (for technical details see Materials and methods). Note that cell lines 530 and 1F6 did not proliferate when seeded at low density

**Figure 4** Growth curves of s.c. melanoma xenografts in vivo. Each point represents the volume of a single xenograft measured at autopsy. Non-linear regression analysis was performed for each data set of malignancy (in vivo growth rate, ability to form spontaneous lung metastases), and the expression level of VPF.

### In vitro and in vivo growth rate of melanoma cell lines

The melanoma xenografts displayed different in vivo growth rates. To investigate whether this could be attributed to an intrinsic proliferation rate of the cell lines, or that environmental factors were involved as well, we compared the in vitro and in vivo growth rates of cell lines and xenografts respectively. The in vitro growth rate was determined in the MTT assay with different starting cell densities. Figure 3A shows that 530 and 1F6 cells did not grow when seeded at low density (1500 cells cm$^{-2}$). 1F6m started to grow after an initial lag period, whereas the other cell lines showed similar proliferation rates. When cells were seeded at a higher density (6000 cells cm$^{-2}$), all cell lines showed growth, but 530 and 1F6 grew markedly slower than the other cell lines. 1F6m proliferated faster than 530 and 1F6, but not as fast as the other lines.

The in vivo growth rate was determined by estimating the tumour volume at the day of autopsy. The highly vascularized BLM and MV3 xenografts showed by far the quickest expansion (Figure 4). After a maximum of 6 weeks, the xenografts had grown to a size when killing of the mice could no longer be postponed. A total of 530 xenografts grew relatively slowly. Most tumours were still less than 1 cm$^3$, 2–3 months after take of the tumour. Xenografts of the other cell lines showed intermediate growth rates. Remarkably, 1F6 xenografts reached larger volumes than 1F6m xenografts, despite the fact that 1F6 showed a lower in vitro proliferation rate. In the category of BLM and MV3 xenografts, no correlation was found between MVD and tumour volume or age (data not shown).

### Formation of lung metastases by melanoma xenografts

The occurrence of lung metastases was determined by screening two tissue sections of lung cross-sections, one at one-third and one at two-thirds of the height of the lungs. Examination of a larger number of tissue sections from more levels of the lungs had shown that > 95% of metastasis-positive lungs were detected in this way

© Cancer Research Campaign 1997

**Figure 5** Analysis of lung (micro)metastasis formation in xenograft-bearing mice. At different time points after tumour take, mice were killed and the lungs were examined microscopically for the presence of lung (micro)metastasis. Note that in mice bearing 530 or 1F6 xenografts hardly any metastatic lesions were found in the lungs, whereas mice with metastases were numerous in the other groups. The presence of metastasis could be detected as early as 2 weeks (BLM) after tumour take. Number of mice without (□) and with (■) lung (micro)metastases

**Figure 6** Example of quantitative determination of VPF mRNA levels in BLM. (**A**) Products of RT-PCR performed with 1 µg of BLM RNA and increasing amounts of competitor RNA. Products were run on a 1% agarose gel and stained with ethidium bromide. Products of 670, 610 and 500 bp are derived from wild-type VPF mRNA (wRNA), the product of 350 bp is derived from competitor RNA (cRNA). The concentration of cRNA increases from left to right, with a fixed amount of sample RNA, resulting in an increasing amount of amplified cRNA accompanied by a decreasing amount of amplified w-VPF mRNA. Each measurement was performed in duplicate. (**B**) log(cRNA concentration) plotted against log(ratio band intensities of wRNA products/band intensity cRNA product). VPF mRNA concentration in samples can be extrapolated from the plot at log(ratio) = 0. (**C**) Results of quantitative VPF mRNA determinations. Two batches of RNA of each cell line were tested, each batch was tested twice. RNA batch 1, □; RNA batch 2, ■

(data not shown). Both the number of metastases and the area of the metastases relative to the area of the lungs were determined. In all eight xenograft types, a large range was observed both in the number of metastases per lung and in the size of the metastases. No evident differences between number of metastases or metastatic burden could be observed between mice carrying xenografts derived from different cell lines (data not shown). Also, no correlation was found between tumour age and metastatic burden in any investigated cell line. It should be noted that most lung metastases were not detectable macroscopically.

Although number and size of metastases were similar among the various groups, we did observe differences in the time points after tumour take at which lung metastases were first observed. Figure 5 shows the occurrence of lung metastasis in mice carrying xenografts of different ages. As early as 10 days after take of the s.c. tumour, micrometastases could be found in mice bearing BLM xenografts. When the tumours had reached their maximal volume, in 60–70% of these mice lung metastases were found. Although metastases formation was somewhat slower in mice carrying xenografts from 1F6m, Mel57 or M14, the percentage of metastasis-positive mice eventually became as high as, or even higher than, that of mice carrying BLM or MV3 xenografts. In the case of Mel57, up to 100% of mice were found to have lung metastases when killed 8 weeks after tumour take. Mice carrying 530 or 1F6 xenografts only sporadically (1 out of 51 and 3 out of 67 respectively) developed lung metastases. In these four mice, the

© Cancer Research Campaign 1997

metastatic burden was very low; only one or two micrometastases per section and never more than ten tumour cells per metastatic lesion were observed.

We looked for a possible correlation between the vascular density of BLM and MV3 xenografts and the presence or absence of metastases or the metastatic burden. No statisticaly significant differences could be observed between MVD values in xenografts of mice without or with lung metastases (Figure 2). Also, no correlation was found between MVD and metastatic burden (data not shown).

### Quantification of VPF mRNA in melanoma cell lines

In a previous study, we showed by Northern blot analysis that VPF is expressed by cell lines BLM and MV3, but not by cell lines 1F6 and Mel57 (Pötgens et al, 1995*b*). When performing an RT-PCR to detect VPF mRNA, positivity was found in RNA samples from all cell lines (data not shown), indicating that VPF mRNA was expressed by all cell lines but in some cell lines was apparently below the detection level of Northern blot. To substantiate these findings, we developed a quantitative RT-PCR for VPF mRNA. Figure 6A shows the products of a series of RT-PCRs after separation on agarose gel and staining with ethidium bromide. After measuring the intensities of the bands, log (concentration competitor RNA) was plotted against log (ratio of band intensities) (Figure 6B). Only experiments with a correlation coefficient of the resulting graph > 0.95 were used. The amount of wild-type VPF mRNA in the sample RNA could be extrapolated by determining the RNA concentration at log (ratio of band intensities) = 0 (Figure 6B). In Figure 6C, results of mRNA concentration determination for two separate experiments are shown. The amount of mRNA in the highly vascularized cell line MV3 was significantly higher than in 1F6, 1F6m, Mel57, M14 ($P < 0.001$) and BLM ($P < 0.05$). VPF mRNA concentrations in BLM were significantly higher compared with those in 1F6, 1F6m, M14 and Mel57 ($P < 0.05$).

### DISCUSSION

In this paper we elaborate on a previously described xenograft model in which human melanoma cells, propagated as cell lines in vitro, are injected subcutaneously into nude mice.

We have measured the vascular densities of xenografted s.c. melanoma tumours. As opposed to most other reports on quantification of the tumour vasculature, we have used automated image analysis of a relatively large tumour area instead of manually selecting one or a few microscopic fields with the highest vascular density (the hotspot' method) as a parameter for determining the vascularity of our tumours. Although we have found (van der Laak et al, submitted for publication) that in our model there is a good correlation between the results of both methods, there are three reasons for using automated image analysis of a large tumour area instead of hot spot density measurements. First, automated image analysis allows one to include vessel parameters, such as vessel diameter in the analysis of the obtained data. As we have shown, vessels of a certain diameter (corresponding by morphological criteria to capillaries), discriminated best between the different groups of xenografts. Second, when assessing the effect of vascularization on the tumour growth rate, the vascularity of the entire tumour (the MVD) will contribute and has to be taken into account. Third, angiogenic hot spots may be more objectively determined by image analysis than by manual selection (van der Laak et al, submitted for publication). Angiogenic hot spots in a tumour may be decisive in determining the metastatic behaviour, as only part of the tumour has to be highly vascularized to increase the probability of metastasizing tumour cells to enter the bloodstream, especially if tumour cells with a metastasizing phenotype are located within this hotspot.

We found that, in BLM and MV3 xenografts, MVD values of vessels between 3 and 12 µm were 2–4 times higher than those of the other cell lines. Analysing subclasses of vessels based on their diameter distinctly enhanced the differentiation into different degrees of vascularization. M14 showed a distinctively lower vessel density than BLM and MV3 in all investigated vessel diameter classes. In the case of Mel57, the difference was most apparent in the 3–6 µm class, whereas 1F6 and MV1 showed the most pronounced differences in the 6–12 µm class.

BLM and MV3 distinguished themselves from the other cell lines in displaying a higher in vivo growth rate, and BLM by formation of spontaneous lung metastases soon after take of the s.c. tumour (8–15 days). The number and size of the lung metastases formed in mice with M14 or Mel57 xenografts were similar to those found in mice carrying BLM xenografts, but they appeared later after tumour take. Lack of correlation between vessel density and metastatic potential was also demonstrated by xenografts derived from 1F6 and its spontaneous descendant 1F6m. These xenografts display vascular profiles that are quantitatively and qualitatively identical, but in 1F6m-bearing mice numerous spontaneous lung metastases were observed, whereas 1F6 xenografts only sporadically metastasized. We conclude that in our model system a high MVD correlates with an increased rate of in vivo proliferation and with faster metastasis formation, but not with the event of metastasis formation per se. Whether the rapid metastasis formation in BLM and MV3 xenografts is a direct consequence of the high vessel number cannot be concluded from our data. Obviously, the rapid growth rate of the xenografts results in a higher number of cells that have the opportunity to metastasize. Alternatively, the intrinsic properties of different types of tumour cells may be the cause of different metastasis formation speeds. Previous studies have shown that BLM and MV3 differ from the other cell lines with respect to factors that influence the metastatic process. They show a strong adherence to extracellular matrix components laminin and collagen types I and IV (Danen et al, 1993, 1995), and have a high expression level of the extracellular protease urokinase-type plasminogen activator (uPA) and its inhibitor PAI-1 (Quax et al, 1991; Van Muijen et al, 1995). The formation of experimental lung metastasis may be used as an indicator of the metastasizing capacities of a cell line. This capacity was investigated by van Muijen et al (1991*a*) for cell lines 530, 1F6, M14, Mel57, MV3 and BLM. All lines, with the exception of 1F6, were able to form experimental lung metastases with an efficiency ranging between 50% and 90%, with the highest percentages found in mice injected with BLM or MV3. This superior intrinsic metastatic capacity of circulating BLM and MV3 cells may have contributed to the higher speed of metastasis formation we observed for these cell lines.

The relationship between vascular density and malignancy in human melanoma is currently under debate. Whereas preliminary studies indicated that microvessel density in these tumours correlated with their potential to invade and metastasize (Srivastava et al, 1988; Barnhill et al, 1992; Graham et al, 1994), other reports could not confirm this (Carnochan et al, 1991; Barnhill et al, 1994; Busam et al, 1995). In our model system, there was no correlation between vascular density and the ability to metastasize as well.

© Cancer Research Campaign 1997

One should bear in mind, however, that s.c. human tumours that are grown in nude mice are not fully comparable to human primary melanomas with respect to location (subcutaneous vs intra(epi)dermal), origin (inoculation with a bulk of cultured cells vs mono- or oligoclonal expansion) and source of the vascular bed (mouse vs human).

Our current and previous data (Pötgens et al, 1995*a*) have shown that BLM and MV3 show an expression level of VPF protein and mRNA that is clearly elevated compared with the cell lines that generate poorly vascularized tumours, emphasizing the previously described role (Folkman and Klagsbrun, 1987; Leung et al, 1989; Klagsbrun and D'Amore, 1991) of this factor in the angiogenic process. Pötgens et al (1995*b*, 1996) transfected the low VPF-producing cell line Me157 with the $VPF^{121}$ gene, resulting in a cell line with an in vitro expression level of this VPF isoform that was 1000-fold higher than in the parental cell line. Xenografts derived from the transfected cell line showed a vascular pattern that was dramatically changed compared with parental cell line xenografts. In the parental xenografts vessels were scattered randomly throughout the tumour, whereas in the mutant xenografts tumour nodules were surrounded by highly vascularized septa. Frequency, size and time point of occurrence of spontaneous lung metastases did not differ in mice bearing either xenograft. Complementary data were recently presented by Claffey et al (1996). Inoculation of mice with melanoma cell line SK-Mel-2 transfected with mouse $VPF^{164}$ cDNA, resulted in highly vascularized tumours with an increased growth rate compared with tumours derived from mock-transfected cells. Moreover, VPF-transfected cells showed an increased ability to form experimental lung metastases after i.v. injection. The effect of transfection with VPF on the formation of spontaneous lung metastases was not assessed in this study. As angiogenesis in unlikely to play a role in the initial take and growth of (micro)metastases, the mechanism by which VPF increased metastatic potential remains unclear. It is possible that the permeabilization effect of VPF on the vessel wall leads to a facilitated extravasation of tumour cells. This possibility, however, does not explain why increased metastatic incidence was not observed in the experiments with VPF-transfected melanoma cell lines described by Pötgens et al (1996), as $VPF^{121}$ is a potent inducer of vessel permeability. Further data on the role of VPF in angiogenesis and metastasis formation come from the observation that VPF mRNA is induced in 1F6 and Me157 xenografts because of hypoxic conditions (Pötgens et al, 1995*b*). This up-regulation, however, did not lead to increased vascular densities in 1F6 and Mel57 xenografts, it did not induce metastasis formation in 1F6 bearing mice and it did not increase speed of metastasis formation in Mel57 mice. It is reasonable to assume that also in 530, 1F6m, M14 and MV1 xenografts, VPF mRNA expression is up-regulated because of hypoxic conditions. In these cell lines no effect on MVD and/or speed of metastases formation was found compared with BLM and MV3. Taken together, these data show that VPF may be required for, and/or augments the vascularized phenotype and the metastatic potential of a melanoma xenograft, but may have to act in concert with one or more other (angiogenic) factors. To shed more light on this issue, we are currently investigating the expression levels of a panel of other angiogenic factors on protein and mRNA level in both the melanoma cell lines and in the corresponding xenografts.

In this paper we have analysed some aspects of tumour angiogenesis in an in vivo model for human melanoma. Although in this model a vascular bed of murine origin is generated by human angiogenic factors, rapid tumour growth and metastatic spread occur, apparently unhindered by the species barrier, thereby confirming the suitability of the model to study the process of angiogenesis. Both the vascularity of the xenografts and the metastatic burden can be easily quantified in a standardized manner. Considering the different vascular densities we found in xenografts derived from the different cell lines, this model provides us with material that is well suited to investigate the mechanisms that are responsible for this increased vascular density. Furthermore, our model may allow for testing the in vivo effects of antiangiogenic drugs on tumour growth and vascular densities in a preclinical setting.

### ABBREVIATIONS

bFGF, basic fibroblast growth factor; EC, endothelial cell; MAb, monoclonal antibody; MVD, mean vascular density; RT-PCR, reverse transcriptase-polymerase chain reaction; VPF/VEGF, vascular permeability factor/vascular endothelial growth factor.

### ACKNOWLEDGEMENTS

The authors wish to thank Bert Smeets and Willy Nillesen (Department of Human Genetics, University Hospital Nijmegen, Nijmegen) for performing CA repeat analysis, Coos Diepenbroek and co-workers for excellent histological analysis, and Dr A Hamann and Dr A Vecchi for providing the 9F1 hybridoma and the MEC7.46 antibody respectively. This work was supported by the Dutch Cancer Society (Grant NUKC 92-36).

### REFERENCES

Albelda SM (1993) Biology of disease. Role of integrins and other cell adhesion molecules in tumor progression and metastasis. *Lab Invest* **68**: 4–17

Barnhill RL, Fandrey K, Levy MA, Mihm MC, Jr and Hyman B (1992) Angiogenesis and tumor progression of melanoma. Quantification of vascularity in melanocytic nevi and cutaneous malignant melanoma. *Lab Invest* **67**: 331–337

Barnhill RL, Busam KJ, Berwick M, Blessing K, Cochran AJ, Elder DE, Fandrey K, Karaoli T and White WL (1994) Tumour vascularity is not a prognostic factor for cutaneous melanoma. *Lancet* **344**: 1237–1238

Bigler SA, Deering RE and Brawer MK (1993) Comparison of microscopic vascularity in benign and malignant prostate tissue. *Hum Pathol* **24**: 220–226

Busam KJ, Berwick M, Blessing K, Fandrey K, Kang S, Karaoli T, Fine J, Cochran AJ, White WL, Rivers J, Elder DE, Po Wen D-R, Heyman BH and Barnhill RL (1995) Tumor vascularity is not a prognostic factor for malignant melanoma of the skin. *Am J Pathol* **147**: 1049–1056

Carnochan P, Briggs J, Westbury G and Davies A (1991) The vascularity of cutaneous melanoma: a quantitative histological study of lesions 0.85–1.25 mm in thickness. *Br J Cancer* **64**: 102–107

Claffey KP, Brown LF, Del Aguila LF, Tognazzi K, Yeo KT, Manseau EJ and Dvorak HF (1996) Expression of vascular permeability factor/vascular endothelial growth factor by melanoma cells increases tumor growth, angiogenesis, and experimental metastasis. *Cancer Res* **56**: 172–181

Danen EHJ, Van Muijen GNP, Van De Wiel-Van Kemenade E, Jansen KFJ, Ruiter DJ and Figdor CG (1993) Regulation of integrin-mediated adhesion to laminin and collagen in human melanocytes and in non-metastatic and highly metastatic human melanoma cells. *Int J Cancer* **54**: 315–321

Danen EHJ, Van Muijen GNP and Ruiter DJ (1995) Role of integrins as signal transducing cell adhesion molecules in human cutaneous melanoma. *Cancer Surveys* **24**: 43–65

Folkman J (1971) Tumor angiogenesis: therapeutic implications. *N Engl J Med* **285**: 1182–1186

Folkman J (1995*a*) Clinical applications of research on angiogenesis. *N Engl J Med* **333**: 1757–1763

Folkman J (1995*b*) Angiogenesis in cancer, vascular, rheumatoid and other disease. *Nature Medicine* **1**: 27–31

© Cancer Research Campaign 1997

Folkman J and Klagsbrun M (1987) Angiogenic factors. *Science* **235**: 442–447

Folkman J and Shing Y (1992) Angiogenesis. *J Biol Chem* **267**: 10931–10934

Graham CH, Rivers J, Kerbel RS, Stankiewicz KS and White WL (1994) Extent of vascularization as a prognostic indicator in thin (< 0.76 mm) malignant melanomas. *Am J Pathol* **145**: 510–514

Horak ER, Leek R, Klenk N, Lejeune S, Smith K, Stuart N, Greenall M, Stepniewska K and Harris AL (1992) Angiogenesis, assessed by platelet/endothelial cell adhesion molecule antibodies, as indicator of node metastases and survival in breast cancer. *Lancet* **340**: 1120–1124

Klagsbrun M and D'Amore PA (1991) Regulators of angiogenesis. *Annu Rev Physiol* **53**: 217–239

Leung DW, Cachianes G, Kuang W-J, Goeddel DV and Ferrara N (1989) Vascular Endothelial Growth Factor is a secreted angiogenic mitogen. *Science* **246**: 1306–1309

Liotta LA, Steeg PS and Stetler Stevenson WG (1991) Cancer metastasis and angiogenesis: an imbalance of positive and negative regulation. *Cell* **64**: 327–336

Macchiarini P, Fontanini G, Hardin MJ, Squartini F and Angeletti CA (1992) Relation of neovascularisation to metastasis of non-small-cell lung cancer. *Lancet* **340**: 145–146

Nelen MR, Van Der Burgt CJAM, Nillesen WN, Vis A and Smeets HJM (1994) Familial Angelman syndrome with a crossover in the critical deletion region. *Am J Med Gen* **52**: 352–357

Pötgens AJG, Westphal JR, De Waal RMW and Ruiter DJ (1995*a*) The role of vascular permeability factor and basic fibroblast growth factor in tumour angiogenesis. *Biol Chem Hoppe-Seyler* **376**: 57–70

Pötgens AJG, Lubsen NH, Van Altena MC, Schoenmakers JGG, Ruiter DJ and De Wall RMW (1995*b*) Vascular permeability factor expression influences tumor angiogenesis in human melanoma lines xenografted to nude mice. *Am J Pathol* **146**: 197–209

Pötgens AJG, Van Altena MC, Lubsen NH, Ruiter DJ and De Waal RMW (1996) Analysis of the tumor vasculature and metastatic behaviour of xenografts of human melanoma cell lines transfected with vascular permeability factor. *Am J Pathol* **148**: 1203–1217

Quax PHA, Van Muijen GNP, Weening-Verhoeff EJD, Lund LR, Dano K, Ruiter DJ and Verheijen JH (1991) Metastatic behaviour of human melanoma cell lines in nude mice correlates with urokinase-type plasminogen activator, its type-1 inhibitor, and urokinase-mediated matrix degradation. *J Cell Biol* **115**: 191–199

Smolle J, Soyer H-P, Hofmann-Wellenhof R, Smolle-Juettner F-M and Kerl H (1989) Vascular architecture of melanotic skin tumors. A quantitative immunohistochemical study using automated image analysis. *Path Res Pract* **185**: 740–745

Srivastava A, Laidler P, Davies RP, Horgan K and Hughes LE (1988) The prognostic significance of tumor vascularity in intermediate-thickness (0.76–4.0 mm thick) skin melanoma. A quantitative histologic study. *Am J Pathol* **133**: 419–423

Van Muijen GNP, Cornelissen LMAH, Jansen CFJ, Figdor CG, Johnson JP, Bröcker E-B and Ruiter DJ (1991*a*) Antigen expression of metastasizing and non-metastasizing human melanoma cells xenografted into nude mice. *Clin Exp Metastasis* **9**: 259–272

Van Muijen GNP, Jansen CFJ, Cornelissen IMAH, Smeets DFCM, Beck JLM and Ruiter DJ (1991*b*) Establishment and characterization of a human melanoma cell line (MV3) which is highly metastatic in nude mice. *Int J Cancer* **48**: 85–91

Van Muijen GNP, Danen EHJ, De Vries TJ, Quax PHA, Verheijen JH and Ruiter DJ (1995) Properties of metastasizing and nonmetastasizing human melanoma cells. *Recent Results Cancer Res* **139**: 105–122

Vecchi A, Garlanda C, Lampugnani MG, Resnati M, Matteuci C, Stoppacciaro A, Schnurch H, Risau W, Ruco L, Mantovani A and Dejana E (1994) MAb specific for endothelial cells of mouse blood vessels. Their application in the identification of adult and embryonic endothelium. *Eur J Cell Biol* **63**: 247–254

Wakui S, Furusato M, Itoh T, Sasaki H, Akiyama A, Kinoshita I, Asano K, Tokuda T, Aizawa S and Ushigome S (1992) Tumour angiogenesis in prostatic carcinoma with and without bone marrow metastasis: a morphometric study. *J Pathol* **168**: 257–262

Weidner N, (1995) Intratumor microvessel density as a prognostic factor in cancer. *Am J Pathol* **147**: 9–19

Weidner N, Semple JP, Welch WR and Folkman J (1991) Tumor angiogenesis and metastasis – correlation in invasive breast carcinoma. *N Engl J Med* **324**: 1–8

Weidner N, Carroll PR, Flax J, Blumenfeld W and Folkman J (1993) Tumor angiogenesis correlates with metastasis in invasive prostate carcinoma. *Am J Pathol* **143**: 401–409

Weinstat Saslow D and Steeg PS (1994) Angiogenesis and colonization in the tumor metastatic process: basic and applied advances. *FASEB J* **8**: 401–407

Weterman MAJ, Stoopen GM, Van Muijen GNP, Kuznicki J, Ruiter DJ and Bloemers HPJ (1992) Expression of calcyclin in human melanoma cell lines correlates with metastatic behaviour in nude mice. *Cancer Res* **52**: 1291–1296

© Cancer Research Campaign 1997